# Intelligent Prediction for Rock Porosity While Drilling Complex Lithology in Real Time

**DOI:** 10.1155/2021/9960478

**Published:** 2021-06-14

**Authors:** Hany Gamal, Salaheldin Elkatatny, Ahmed Alsaihati, Abdulazeez Abdulraheem

**Affiliations:** College of Petroleum Engineering & Geosciences, King Fahd University of Petroleum & Minerals, Dhahran 31261, Saudi Arabia

## Abstract

Rock porosity is an important parameter for the formation evaluation, reservoir modeling, and petroleum reserve estimation. The conventional methods for determining the rock porosity are considered costly and time-consuming operations during the well drilling. This paper aims to predict the rock porosity in real time while drilling complex lithology using machine learning. In this paper, two intelligent models were developed utilizing the random forest (RF) and decision tree (DT) techniques. The drilling parameters include weight on bit, torque, standpipe pressure, drill string rotation speed, rate of penetration, and pump rate. Two datasets were employed for building the models (3767 data points) and for validating the developed models (1676 data points). Both collected datasets have complex lithology of carbonate, sandstone, and shale. Sensitivity and optimization on different parameters for each technique were conducted to ensure optimum prediction. The models' performance was checked by four performance indices which are coefficient of determination (*R*^2^), average absolute percentage error (AAPE), variance account for (VAF), and a20 index. The results indicated the strong porosity prediction capability for the two models. DT model showed *R*^2^ of 0.94 and 0.87 between the predicted and actual porosity values with AAPE of 6.07 and 9% for training and testing, respectively. Generally, RF provided a higher level of strong prediction than DT as RF achieved *R*^2^ of 0.99 and 0.90 with AAPE of 1.5 and 7% for training and testing, respectively. The models' validation proved a high prediction performance as DT achieved *R*^2^ of 0.88 and AAPE of 8.58%, while RF has *R*^2^ of 0.92 and AAPE of 6.5%.

## 1. Introduction

The porosity of the rock is commonly defined as the ratio between the void pore spaces in the rock to the total bulk volume for the rock, and this space will provide the storage capacity for the petroleum fluids if it is connected. The rock porosity is a vital petrophysical property as it has a great impact on the reservoir reserve estimation, and as a result, for the field development decision-making [1, 2]. The precise determination of the rock porosity will significantly affect the petroleum reserve estimation and economics [3], and hence, the accuracy of the porosity determination will play a huge role.

Determining the rock porosity can be achieved practically by direct and indirect measurement or prediction using empirical equations. Laboratory measurements for the cored rock samples are the direct way to measure the rock porosity [4]; however, determining the porosity from the porosity logs is considered an indirect way to acquire the porosity values [5]. Each technique has its pros and cons from technical and economic aspects as the lab direct measurements for the porosity is considered the most relative accurate way; however, this technique is costly and time-consuming and covers only the cored interval within the reservoir or the drilled sections [6, 7]. On the contrary, the porosity determination by the well logging tools has many types of error as the operational calibration for the logging tool, in addition to the borehole and mud impact on the measurement [8, 9]. A recent technique is introduced to the field applications of rock characterization and rock porosity measurement by employing the drilled cuttings; however, the technique required special cuttings size and advanced sample preparation [10].

Determining the rock porosity from the well logs data approach was studied in the literature where the rock porosity was obtained based on other petrophysical logs [11, 12]. Nuclear magnetic resonance measurement was introduced for determining the rock porosity [13]. However, such techniques required the logging data or lab measurements to determine the rock porosity values that required extra cost and time.

The applications of artificial intelligence (AI) techniques provided huge contributions for dealing with petroleum data in different disciplines. AI tools such as artificial neural networks (ANNs), fuzzy logic (FL), expert systems, support vector machines (SVMs), functional networks (FN), and case-based reasoning provided high performance and accurate prediction results [14]. The implementation of such tools contributed to solving many technical problems such as estimation and optimization of drilling parameters [15–19], predicting and monitoring the drilling fluids properties [20–24], reservoir fluid properties [25–30], rock permeability estimation [31, 32], and rock strength and geomechanical properties [33–37]. These applications provided different approaches in terms of the AI techniques and the input parameters.

The porosity prediction by employing artificial intelligence techniques was studied in the literature as shown in Table 1. The table shows the input parameters for predicting the rock porosity, data points, rock formation type for the study, correlation coefficient (*R*) between the predicted and the actual porosity values, and the AI techniques that were employed to build the prediction models.

The studies investigated the core porosity prediction using the well logging data as density, neutron porosity, sonic time, resistivity log, gamma-ray (GR), and stratigraphic information [38–40]. In addition, the drilling data was employed for predicting the formation porosity using drilling parameters as the rate of penetration (ROP), pump rate (Q), drill string rotating speed (RPM), standpipe pressure (SPP), torque (T), weight on bit (WOB), and mechanical specific energy [41, 42].

As shown in the literature, drilling data was employed but for carbonate formation during drilling horizontal well [41], and another study for sandstone and shale formations but with incorporating the mechanical specific energy as an additional input to the drilling data; furthermore, the model accuracy was low with a correlation coefficient between the predicted and actual porosity values of 0.6 [42]. The novel contributions for this research are generating the formation neutron porosity log from only the available surface drilling parameters for complex lithology drilled rocks with high accuracy using AI models. The current study predicted the porosity using a collected drilling data during drilling complex lithology formations that have carbonate, sand, and shale formations. The study introduced two AI models for the porosity prediction using decision tree (DT) and random forest (RF) tools. The obtained models from this study will help to save the operational cost and time to log or measure the rock porosity in the lab.

## 2. Materials and Methods

**Figure 1:**
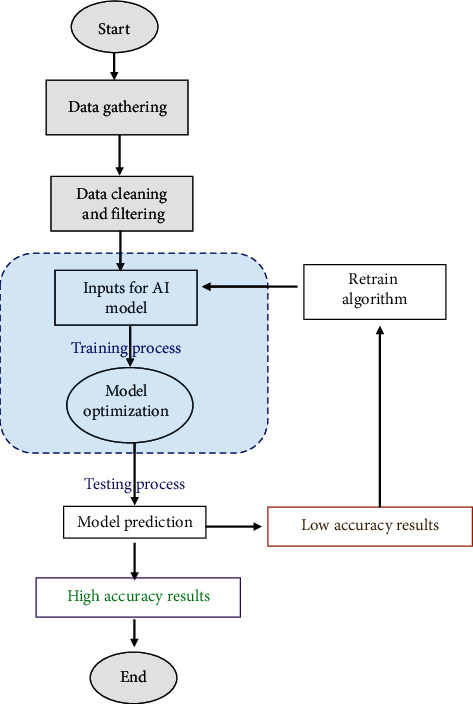
Methodology layout for building AI models.

This research proposed two prediction models for the rock porosity using the drilling data as inputs. The study employed ANN and RF as AI tools for building the prediction model. Using these models, the porosity profile was generated with high accuracy for the whole drilled section that contains complex formation.  Figure 1 represents the processing flow to provide robust models for rock porosity prediction. The data gathering and preprocessing include collecting the data from the drilling sensors and log data for data cleaning to remove the illogic values and outliers. The next step is to build the AI model structure and optimize the model parameters and learning algorithms in order to have good prediction results. The model went through training and testing processes and the results were checked by the statistical performance indices and if the accuracy level of the results is not high enough; then the model should be retrained for enhancing the accuracy level. By the end, the best model parameters and learning algorithm should be saved and reported.

### 2.1. Data Description and Statistics

The data in this study was collected during a drilling phase that covered the intermediate section for vertical wells. The drilled formations contain more than one rock type as sandstone, shale, and limestone that can be considered complex lithologies. The data covered 3767 readings for all the drilling parameters with the neutron porosity log after the data cleaning and preprocessing was used for building the machine learning models. Another dataset of 1670 data points was collected from the same drilling phase that was employed for validating the developed models. The drilling parameters include the surface drilling parameters as the weight on bit (WOB) in klb, torque (T) in kft.lbf, standpipe pressure (SPP) in psi, drill string rotary speed (RPM) in min^−1^, drilling rate of penetration (ROP) in ft/h, and mudflow rate (Q) in gpm.

### 2.2. Data Quality, Preprocessing, and Statistics

The collected data from the drilling sensors suffered from operational measurement and tool errors. And hence, the data should be preprocessed for removing the missing measurements, noise, and outliers by using a developed MATLAB code to ensure the data quality for developing the AI models.

Statistical analysis for the cleaned data shows the minimum, maximum, mean, standard deviation, kurtosis, skewness, and the data range for each parameter as shown in Table 2.

**Figure 2:**
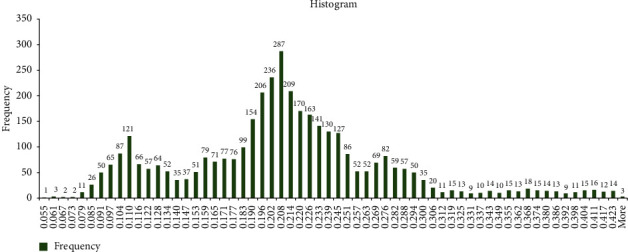
Histogram for the recorded porosity data.

From the data statistics, the drilling parameters and porosity indicated the wide range for the data that will enhance the prediction capabilities of the developed AI models. The statistics show that WOB ranged from 1.5 to 26.7 (klbf), T from 4.3 to 11.0 (kft.lbf), SPP from 2140 to 3076 psi, pipe speed from 77.9 to 162.5 (1/min), ROP from 26.1 to 119.6 (ft/h), flow rate ranged from 627 to 854 (gpm), and the target parameter from 0.055 to 0.429 that covered very tight rock class to high porous rock scale.  Figure 2 represents the porosity histogram for the recorded value. The histogram shows that the frequency of the porosity profile changed with the recorded porosity; the porosity values below 0.2 recorded 44% of the total recorded frequency, 49% of the total frequencies were recorded for the porosity values from 0.2 to 0.3, and only 8% from the total frequency was observed for the higher porosity values greater than 0.3. Hence, the porosity database covered a wide range for the rock porosity data that enhances the capability of the prediction models.

**Figure 3:**
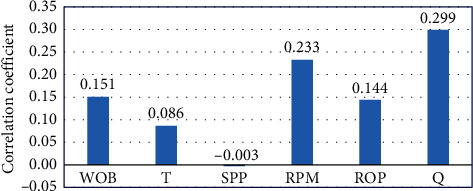
Correlation coefficient of drilling parameters with rock porosity.

The relationships between the drilling parameters (model inputs) and the rock porosity (model output) show a direct linear relationship between the porosity and drilling parameters as Q, RPM, WOB, ROP, and T with a correlation coefficient (*R*) of 0.299, 0.233, 0.151, 0.144, and 0.086, respectively. However, the porosity shows a very weak indirect relationship with SPP by *R* of −0.003. As represented in Figure 3. However, it worthly mentioned that the relationship between the porosity and drilling parameters might reveal a nonlinear relationship.

### 2.3. Building and Evaluating the Artificial Intelligent Models

Two techniques were implemented in this study for building rock porosity models which are decision tree and random forest as they have the same tree-based technique. These techniques are well known and applied in many petroleum studies [43].

**Figure 4:**
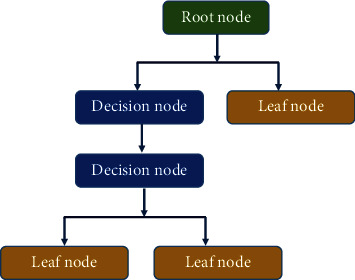
Schematic for the decision tree and its parameters.

Decision tree (DT) is considered one of the AI algorithms and it is a simple approach for application [44]. The technique employed straightforward rules for decision-making based on inferred decision instructions [45]. The technique has a hierarchical construction that contains root node, decision nodes, leaf nodes, and branches as shown in Figure 4. Optimizing these parameters will lead to enhancing the model performance for better prediction [46].

**Figure 5:**
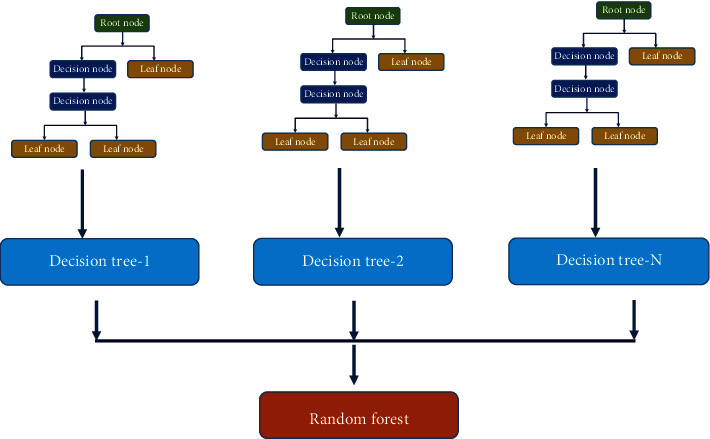
Schematic for the random forest structure.

Random forest is another supervised machine learning algorithm for classification and regression purposes. RF was introduced in 1995 and had several modifications over time [47–50]; it is designed to overcome the overfitting that usually happens in classical discussion trees [51]. Similar to the other machine learning algorithms, several applications of random forest in the oil industry have been reported for classification [52] and regression [53–55].  Figure 5 represents the layout for the random forest as it includes a number N of decision trees in the model structure.

The developed models were evaluated by determining four statistical parameters which are correlation coefficient of determination (*R*^2^), average absolute percentage error (AAPE), variance account for (VAF), and a20 index. These parameters are calculated as follows:

(1) $$\begin{array}{c} R^{2}=\;\frac{\sum_{i}^{N}\left({\hat{y}}_{i}-y_{i}\right)}{\sum_{i}^{N}\left({\hat{y}}_{i}-\;\bar{y}\right)},\\ \text{AAPE}=\left(\frac{1}{N}\overset{N}{\underset{i=1}{\sum }}\left|\frac{y_{i}-{\hat{y}}_{i}}{y_{i}}\right|\times 100\right),\\ \text{VAF}=\left[1-\frac{\text{VAR }\left(y_{i}-{\hat{y}}_{i}\right)}{\text{VAR}\left(y_{i}\right)}\right],\\ \text{a}20\text{index}=\frac{m20}{N}, \end{array}$$

where *N* is the number of data points in the dataset, *y*_*i*_ is the actual output, ${\hat{y}}_{i}$ is the predicted output, $\bar{y}$ is the mean value, and m20 is the number of data points that have ($\;0.8*y_{i}<\hat{y}i\;<1.2*y_{i}$).

## 3. Results and Discussion

**Figure 6:**
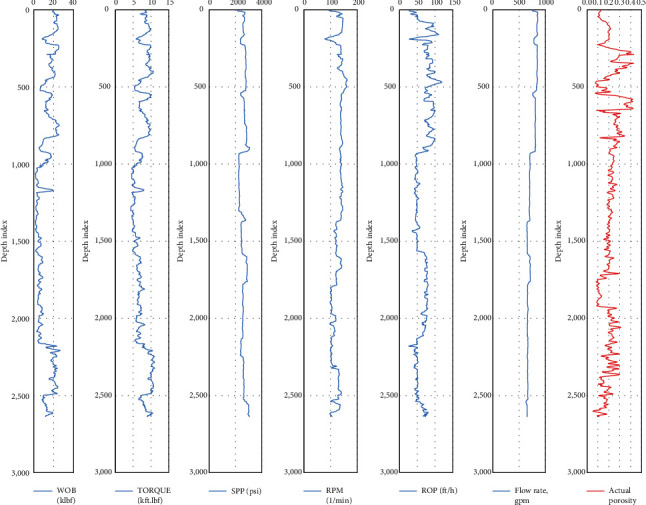
Plotting the input variables (drilling data) versus the output (porosity).

The cleaned data after the preprocessing phase is plotted as shown in Figure 6 to illustrate the problem complexity for modeling the porosity prediction from the drilling data. The boundaries of the drilling parameters in addition to the rock porosity have a great impact on the models' performance. The plot represents the boundaries for the training set parameters that are the output (porosity) versus input variables (drilling data).

### 3.1. DT Model

The data was randomly distributed to training and testing sets by 70 : 30% as 2637 data points for training and 1130 points for the testing set from all the model dataset of 3767 recordings. Max_depth, max_features, min_sample _split, and min_sample_leaf are the main parameters for the DT structure, where max_depth is the most effective parameter as it controls the distance between the root and the leaf node and hence has a great impact on the tree growth. Many sensitivity runs were executed to determine the optimum DT model parameters and the best parameters were recorded and are listed in Table 3.

**Figure 7:**
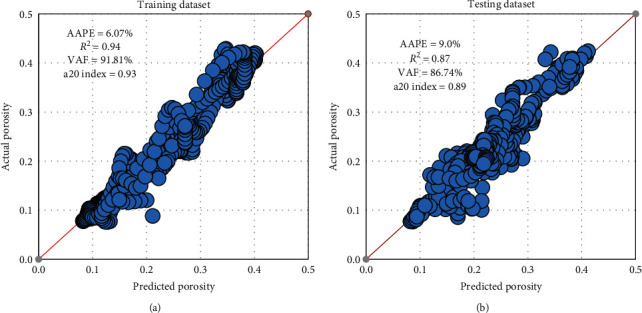
DT model results. (a) Training. (b) Testing.

Figure 7 represents the cross plot for the actual versus the predicted values of the rock porosity for training and testing datasets as AAPE was 6.07% for training and 9% for testing, *R*^2^ was 0.94 and 0.87 for training and testing, respectively. VAF was higher than 86.7% and a20 index greater than 0.89 for training and testing phases.

**Figure 8:**
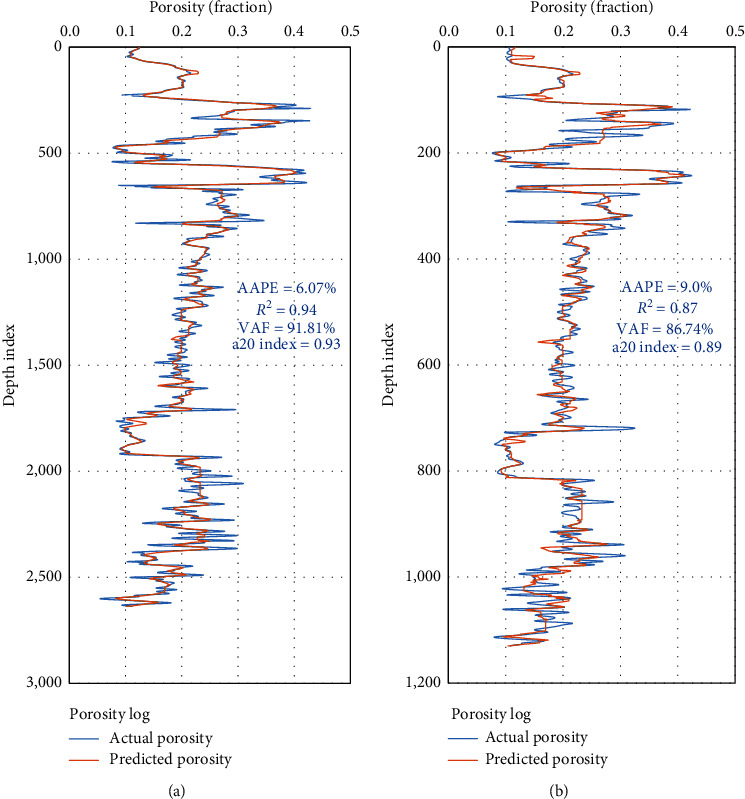
DT porosity model results for the drilled section. (a) Training. (b) Testing.

The obtained results showed a high degree of match between the actual and the predicted values for the porosity profile for the drilled section of different lithology formation types as presented in Figure 8.

### 3.2. RF Model

The same approach was followed to obtain the optimum RF model parameters. The hyperparameters in RF are used either to enhance the predictive power or to make the model faster to run. The n_ estimators parameter is a hyperparameter which is the number of trees that RF builds before computing the average of predictions. A high number of n_estimators enhances the performance of the model and makes the prediction more stable, but it slows down the process of computation. Max_features is another hyperparameter which is the number of features to be considered to split a node in each decision tree. If max_features is “Sqrt”, then the number of features to be considered is the square root of the number of input variables in a dataset. If max_features is “Log2”, then the number of features to be considered is the base-2 logarithm of the number of input variables in a dataset. Max_depth is another important hyperparameter that represents the depth of each tree in a forest. In practice, deep trees can capture more information about the dataset, but also can cause model over-fitting. Therefore, max_depth was tuned from 1 to32 to find the optimum value. Min_samples_split, which represents the minimum number of samples required to split an internal node, and min_samples_leaf, which is the minimum number of samples required to be at a leaf node, are also important hyperparameters for the RF model.  Table 4 shows the optimized RF model parameters.

**Figure 9:**
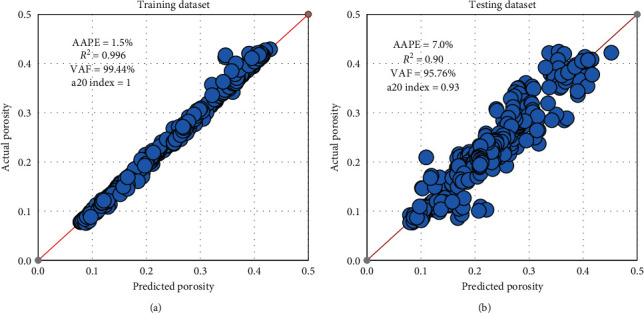
RF model results. (a) Training. (b) Testing.

The training and testing results showed that, for the best RF model parameters, *R*^2^ of 0.99 and 0.90 with AAPE of 1.5 and 7% was observed for the training and testing datasets, respectively, as shown in Figure 9. VAF recorded 99.44% and 95.76%, while the a20 index was 1 and 0.93 for training and testing phases, respectively.

**Figure 10:**
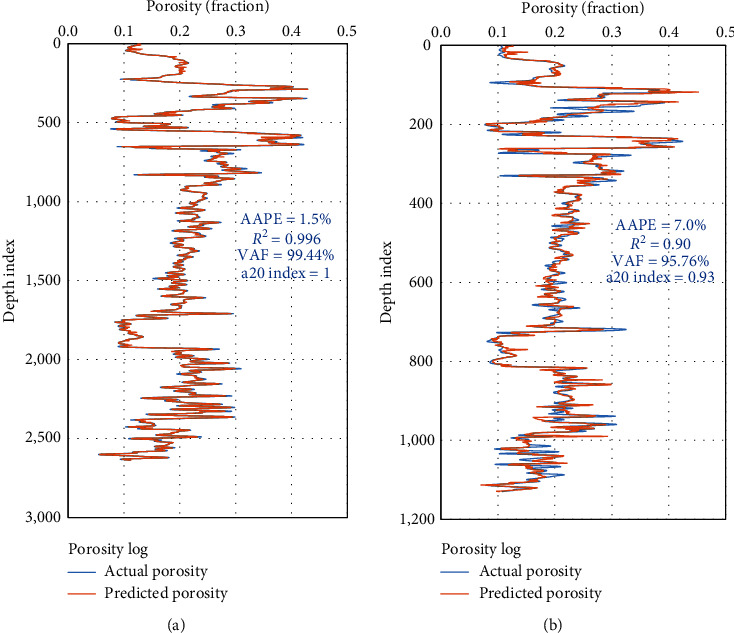
RF porosity model results for the drilled section. (a) Training, (b) testing.

Figure 10 represents the high performance of the RF porosity prediction model for the drilled section. Generally, the two AI-developed models for predicting the rock porosity provided a high level of accuracy during training and testing the model.

### 3.3. Model Validation

**Figure 11:**
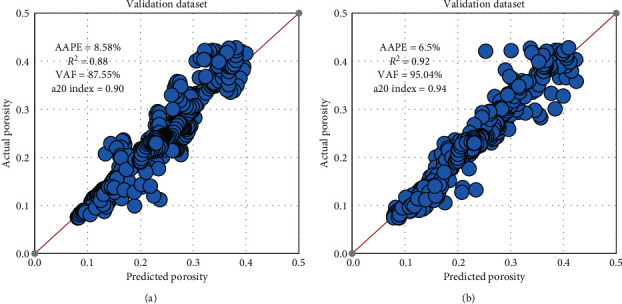
Models validation results. (a) DT. (b) RF.

A different data-set from the same field that has the same penetrated rocks with complex lithology was utilized for validating the developed DT and RF models. This process enhances the practical application for employing the developed model. A cleaned dataset (1670 data points) was employed for validating the models, and the obtained results showed a strong prediction performance for the porosity log from the surface drilling parameters. The validation results showed *R*^2^ of 0.88 and 0.92, and AAPE of 8.58 and 6.5% for DT and RF models, respectively, while VAF and a20 index were higher for RF than DT as shown in Figure 11.

**Figure 12:**
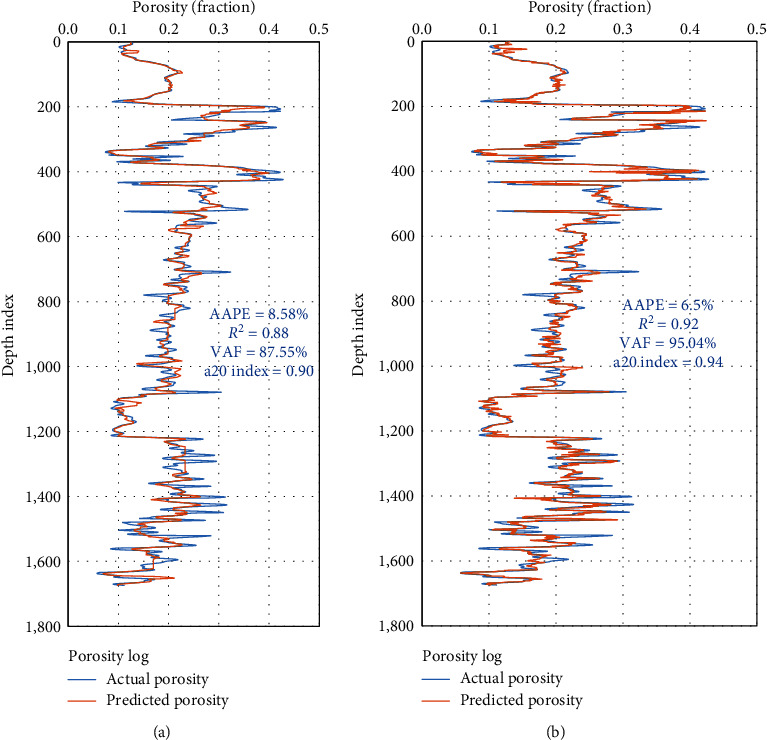
Porosity results for the validation process. (a) ANN model. (b) RF model.

Figure 12 compares the two developed AI models for the validation process. Both DT and RF models are highly performed for predicting the rock porosity for complex lithology formation as shown for the porosity profile of the actual and predicted porosity.

As presented in the results section, the developed RF model showed a great accuracy level in terms of the coefficient of determinations and the average absolute percentage errors between the actual and predicted porosity values. The RF model outperformed the DT with *R*^2^ higher than 0.9 and AAPE less than 7% during the model building and validation phases. In addition, the developed model accuracy is considered a high level compared with the developed model in the literature for predicting the rock porosity; however, the current work targets the complex lithology that means breaking one of the limitations for a specific model for each formation type.

## 4. Conclusions

This study presented a novel approach for predicting the rock porosity from the drilling data during drilling complex lithology formations. The study presented two developed AI models named decision tree (DT) and random forest (RF). The recorded data covered complex lithology of sandstone, shale, and carbonate rock types. Two datasets were employed for building the models and for validating the developed models: a complete dataset of 3763 data points for building the models with a 70 : 30 ratio for training and testing, respectively, while a different dataset (1670 points) for validating the models. The study findings can be summarized as follows:

- DT model parameters were optimized and the results showed *R*^2^ of 0.94 and 0.87 with AAPE of 6.07 and 9% for training and testing, respectively
- The optimized RF model achieved *R*^2^ of 0.99 and 0.90 with an AAPE of 1.5 and 7% for training and testing, respectively
- Validating the two developed models proved the strong prediction performance for the two models with *R*^2^ of 0.88 and 0.92 with AAPE of 8.58 and 6.5% for DT and RF, respectively
- The RF model outperformed the DT for all the key performance indices while training, testing, and validating the models

The porosity estimation in real time will save cost and time for the porosity determination in reality by employing either the lab measurements or well logging operations. The limitations beyond this research can be concluded in the formations type studied in this work, data range for the parameters, and the wellbore geometry as the models were built for intermediate section for vertical wells. So, the study provides recommendations for future work for predicting the rock porosity for complex lithology for other wellbore profiles and drilling sections, in addition to wide range for the data.

## Figures and Tables

**Table 1:** Different correlations for porosity prediction that were developed using AI.

Input parameters	Data points	Formation	*R*	Methods	Ref.
Density, neutron porosity, and sonic compressional time	1700	Carbonate	0.98	ANN, SVM, ANFIS	[38]
Deep resistivity, density, neutron porosity, and gamma-ray	420	Sandstone	0.93	Fuzzy, ANN	[39]
GR, bulk density, resistivity, neutron porosity, and sonic travel time plus lithofacies and stratigraphic information	1000	Carbonate and unconventional	0.98	ANN	[40]
ROP, Q, RPM, SPP, T, WOB	2800	Carbonate	0.96	ANN	[41]
ROP, RPM, WOB, T, depth, SPP, Q, and mechanical specific energy	89549	Sandstone and shale	0.6	ANN	[42]

**Table 2:** Statistical analysis for the cleaned data.

	WOB (klbf)	T (kft.lbf)	SPP (psi)	RPM (1/min)	ROP (ft/h)	Q (gpm)	*ϕ*
Minimum	1.5	4.3	2140.2	77.9	26.1	627.0	0.055
Maximum	26.7	11.0	3076.0	162.5	119.6	854.0	0.429
Mean	11.8	7.4	2600.9	128.5	65.8	724.9	0.207
Standard deviation	7.3	1.8	201.4	15.8	18.2	73.4	0.067
Kurtosis	−1.3	−1.1	−0.7	−0.6	−0.9	−1.3	0.828
Skewness	0.3	0.2	−0.1	−0.5	0.3	0.6	0.552
Range	25.1	6.7	935.8	84.6	93.4	227.0	0.375

**Table 3:** Optimized DT model parameters.

Parameter	Optimum value
Max_depth	10
Max_features	Sqrt
Min_samples_split	2
Min_samples_leaf	1

**Table 4:** Optimized RF model parameters.

Parameter	Optimum value
N_estimators	100
Max_depth	15
Max_features	Sqrt
Min_samples_split	2
Min_samples_leaf	1

## Data Availability

Most of the data are included in the manuscript. For the input data for the model, a sample of the data will be provided upon request.

## Abbreviations

ANN: Artificial neural network
DT: Decision tree
RF: Random forest
SVM: Support vector machines
FL: Fuzzy logic
FN: Functional networks
R2: Coefficient of determination
VAF: Variance account for
AAPE: Average absolute percentage error
AI: Artificial Intelligence
GR: Gamma-ray
WOB: Weight on bit
RPM: Rotating speed in revolutions per minute
ROP: Rate of penetration
Q: Flow rate
SPP: Standpipe pressure
T: Torque.
